# Evidence for long-term seamount-induced chlorophyll enhancements

**DOI:** 10.1038/s41598-020-69564-0

**Published:** 2020-07-29

**Authors:** Astrid B. Leitner, Anna B. Neuheimer, Jeffrey C. Drazen

**Affiliations:** 1 Monterey Bay Aquarium Research Institute, Moss Landing, CA USA; 2 Department of Biological Oceanography, School of Ocean Earth Science and Technology, University of Hawaii at Mānoa, Honolulu, USA; 3 Department of Biology & Aarhus Institute of Advanced Studies, Aarhus University, Århus, Denmark

**Keywords:** Ocean sciences, Biooceanography

## Abstract

Seamounts are ubiquitous global features often characterized by biological hotspots of diversity, biomass, and abundance, though the mechanisms responsible are poorly understood. One controversial explanation suggests seamount-induced chlorophyll enhancements (SICE) subsidize seamount ecosystems. Using a decade of satellite chlorophyll data, we report substantial long-term chlorophyll enhancements around 17% of Pacific seamounts and 45% of shallow (< 100 m) seamounts, with the highest probability of detection at shallow, low-latitude seamounts. SICE is shown to enhance chlorophyll concentrations by up to 56% relative to oceanic conditions, and SICE seamounts have two-fold higher fisheries catch relative to non-enhancing seamounts. Therefore, seamount-induced bottom-up trophic subsidies are not rare, occurring most often at shallow, heavily exploited seamounts, suggesting an important subset of seamounts experience fundamentally different trophic dynamics than previously thought.

## Introduction

Seamounts are submerged mountains that rise at least 1,000 m above the surrounding seafloor^1^. They are one of the most ubiquitous geologic features on our planet with worldwide censuses estimating from tens of thousands up to one hundred thousand seamounts globally, though only a small fraction of these have ever been sampled or even mapped^1,2^. Seamount studies have shown that these features are important ecological habitats which can host diverse and extremely high biomass communities, can play a large role in ocean mixing and global tidal energy dissipation, and can be valuable economic resources in terms of fisheries and minerals^1,3–6^. Even remote seamounts are targets for fisheries, making overfishing a serious concern^6,7^. Seamount ecosystems have also been identified as especially vulnerable to human impacts since they are often characterized by long-lived, slow growing, and fragile animals^6–8^. Moreover, their communities vary greatly in biomass and fishery productivity^4,9^. The mechanisms supporting rich yet vulnerable seamount communities are still poorly understood and highly debated, making conservation and effective management design difficult^8–11^.

It has been proposed that some rich seamount communities are sustained through food subsidies in part from locally enhanced primary production stimulated by increased nutrient availability stemming from seamount-induced current accelerations, upwelling, turbulence, and mixing^10,12,13^. Both modelled and observed circulation patterns suggest that it may be possible that seamounts can increase the amount of chlorophyll in the euphotic zone, and that this could be retained locally^12–15^. Heretofore this effect will be referred to as SICE—seamount induced chlorophyll enhancement. With sufficient magnitude and persistence, SICE could then subsidize both the benthic and pelagic food webs from the bottom up. However, this idea has come under dispute due to contradicting observations from shipboard studies^10,11^, though the limited spatial and temporal resolution of seamount sampling has arguably made it impossible thus far to resolve whether chlorophyll enhancements are commonplace and if they can persist at biologically relevant magnitudes over sufficient timescales to sustain rich and vulnerable seamount communities and their valuable fisheries^7,10^. Here we provide the first spatially expansive, long time-scale test of the disputed hypothesis that seamounts are associated with increased primary producer biomass using a ten-year time series of high resolution satellite ocean color data (oceancolor.gsfc.nasa.gov) in conjunction with two large seamount databases, the Allain database^16^, the largest published and validated seamount database available, and the Yesson database^2^, an unvalidated global seamount database derived from global satellite and gravimetry-based bathymetry (SRMT 30+, see "Methods" for details).

## Results

### Seamount-induced chlorophyll enhancements (SICE) are not rare, and they are more likely at shallow, low latitude seamounts

**Figure 1:**
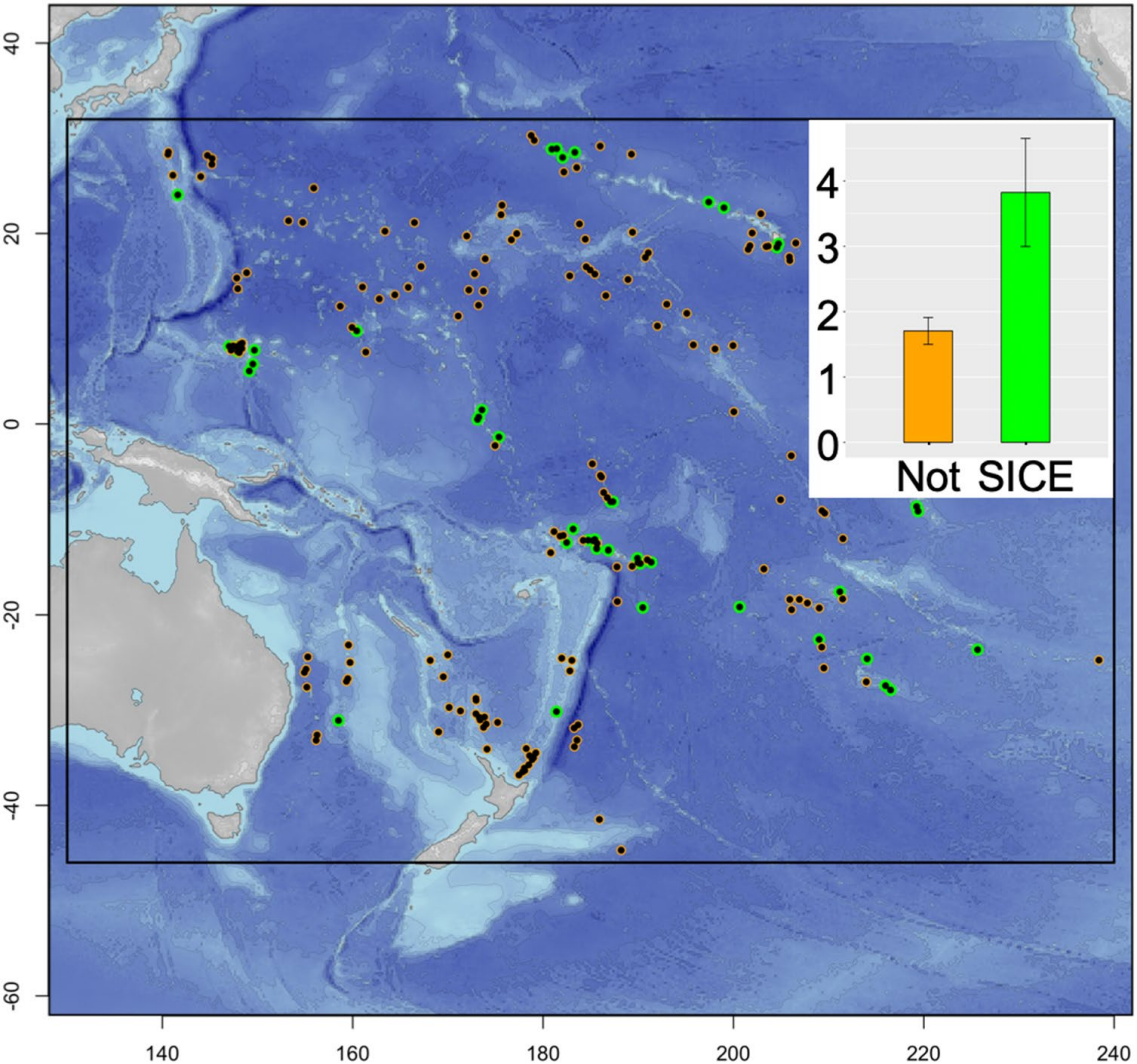
Map of the spatial extent of the validated seamount database used in this study. Black dots represent the seamounts included in the analyses. Green outlines highlight those seamounts with significant sustained chlorophyll enhancements (SICE seamounts, N = 45). Orange differentiates those without (N = 151). Inset shows the significantly higher mean historical total fisheries catch in tens of thousands of tonnes around seamounts with SICE (green bar, mean = 38,245.38 tonnes) as compared to seamounts without SICE (orange bar, mean = 17,050.61 tonnes). Error bars represent the standard error around each mean (*P* < 0.005). Note, means and standard errors include only those seamounts with no emergent pixels within sample region (N = 177).

We have distinguished and categorized SICE and non-SICE seamounts using a generalized additive model (GAM), which, after accounting for spatial and temporal autocorrelation, fit slopes for the relationship between chlorophyll and water depth for each seamount in a representative 177 seamount-subset (see "Methods"), sampled from the Allain et al. database, spanning the western and central Pacific Ocean^16^ (Fig. 1). In addition to being the world’s largest ocean, the Pacific contains nearly half of the world’s seamounts (48%)^17^. A seamount-centered, 100 km-sided square box was analyzed for each seamount. This size encompasses both seamount-influenced water (see "Methods") and unaffected open ocean (control)^18,19^. The statistical significance of these model-fit slopes was used to categorize SICE and non-SICE seamounts. SICE is defined as a positive (increasing chlorophyll with shallower depths), statistically significant (*P* < 0.05) slope (see "Methods"). The model used to identify SICE (vs. pairwise comparison to arbitrary control areas) tests the hypothesis of seamount-enhanced chlorophyll directly, explicitly measuring effects of shallowing bathymetry on chlorophyll concentration while accounting for the natural spatial patchiness of plankton.

About one fifth of all seamounts (17%, 30 of 177) and 45% of seamounts with summits shallower than 100 m could be categorized as SICE seamounts (Fig. 1). If this overall result from the Pacific subset can be extrapolated to global oceans, there could be up to 5,687 SICE seamounts worldwide (17% of an estimated 33,452 global seamounts^2^.

To test the validity of this extrapolation, a randomly stratified, similar-sized subsample (N = 155) of global seamounts was also tested using identical methods (see "Methods"). This global analysis yielded an overall SICE occurrence of 6%, and a 27% SICE occurrence for seamounts with summit depths shallower than 100 m (Table 1). However, our ability to detect SICE at the global scale is hindered by the inaccuracies in the unvalidated global seamount database, which has been shown to contain errors in summit depths and locations, especially at shallow features^16^. Nevertheless, for the global estimate of 1603 seamounts with summit depths shallower than 100 m^2^, results from both global and Pacific subsets still suggest that there are between 433 and 721 (27% and 45% respectively) SICE seamounts globally (Table 1). Regardless of which statistic is used, there are likely hundreds to over 1,000 SICE seamounts in our oceans, making this phenomenon far from rare.

**Table 1:** The summary statistics from the seamount subsets analyzed.

Type	N SICE	Total N seamounts	% SICE
Pacific seamounts (Alllain)	30	177	17
Pacific seamounts plus possible IME	45	196	23
Pacific seamounts $\ge$ − 100 m	10	22	45
Pacific seamounts $\le$ 10° from equator	10	29	34
Pacific seamounts $\ge$ − 500 m	20	60	33
Pacific seamounts $\ge$ − 1,000 m	25	106	24
Global seamounts $\ge$ − 1,000 m (Yesson)	9	155	6
Global seamounts $\ge$ − 100 m	6	22	27
Global seamounts $\le$ 10° from equator	3	26	12

These are summary statistics only, not from model results. Pacific seamounts refer to seamounts from the Allain database. Global Seamounts refer to seamounts extracted from the Yesson database. Seamounts with summit depths shallower than X m are represented as “$\ge -$X m” (e.g. $\ge$ − 100 m). Seamounts with one or more emergent pixels (depth $\ge$ 0 m) within the 100 km sided seamount sample area are "possible Island Mass Effect (IME)" signals. Though all pixels shallower than − 30 m were removed in the data compilation stage, these possible IME seamounts were also flagged and removed before analyses.

Because the proposed mechanism behind SICE is the physical uplift of cooler, deeper, more nutrient rich water, we also examined monthly average SSTs on the same temporal and spatial scales (see "Methods" for details). A spearman’s correlation revealed a significant but weak decrease in temperature with shallower depths overall (rho = − 0.17; *P* < 2.2E−16). However, the relationship between chlorophyll and SST is complex and not monotonic (see Supplementary Information 1 Fig. 1), likely due the presence of both SICE and equatorial upwelling, which gives unexpectedly high chlorophyll values at tropical SSTs. A GAM fitting SST-depth slopes for each seamount revealed that 59% of seamounts (105 of 177) have negative temperature-depth slopes (SST decreases over shallower depths); however, only four slopes were significantly negative. Significant temperature decreases at shallower depths are also positively correlated with SICE (rho = 0.29, *P* = 4.8E−5).

**Figure 2:**
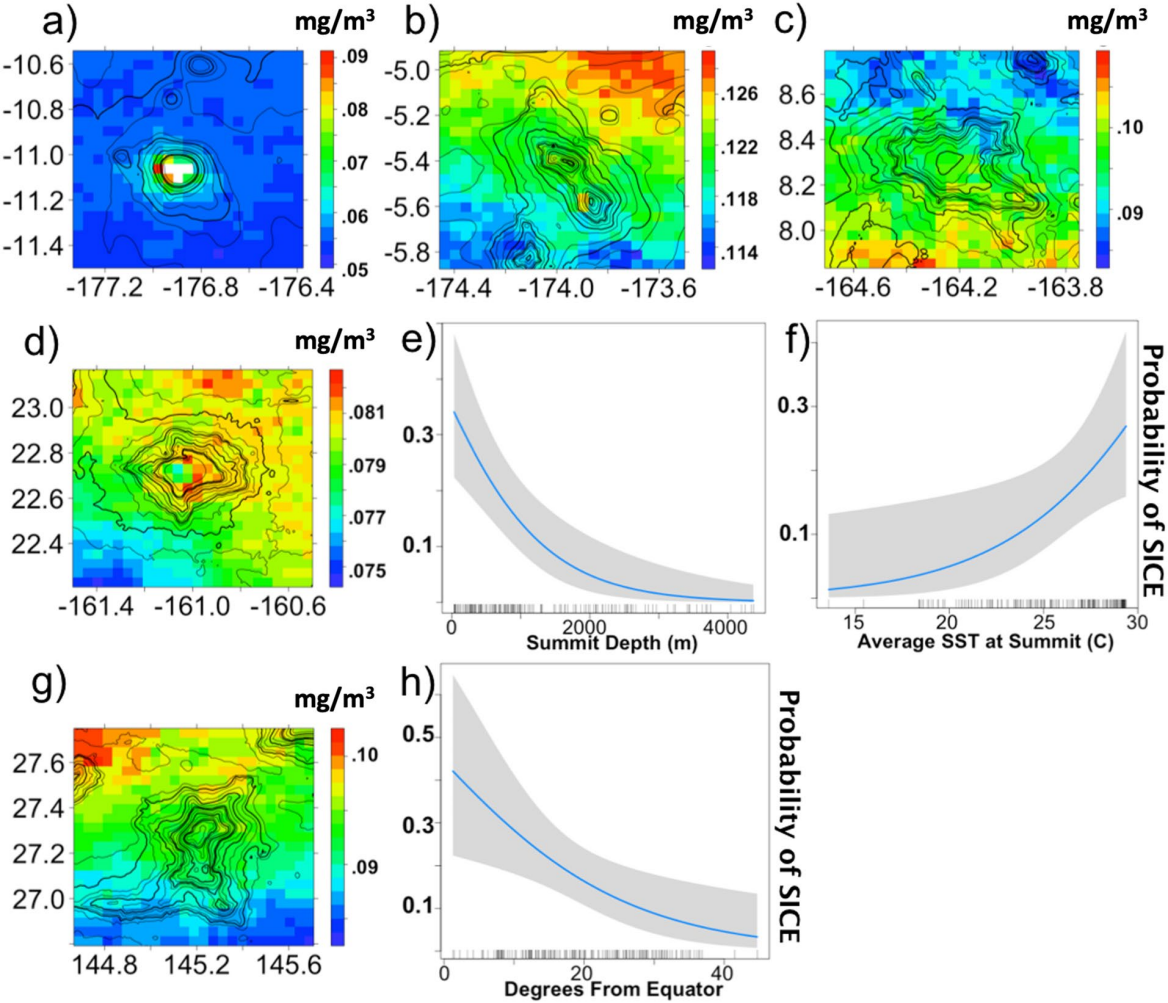
Biogeophysical drivers of seamount-induced chlorophyll enhancements (SICE). Color plots show 10-year average chlorophyll-a concentrations (mg/m^3^) overlain on seamount bathymetry. White pixels have been removed due to the possibility of bias from satellite predicted optically shallow waters (0 m > depth > − 30 m). (**a**–**c**) show seamounts with increasing summit depths (30 m, 315 m, 890 m respectively) while maintaining low latitudes. (**a**, **d**, **g**) show seamounts with increasing latitude (11.0°, 22.7°, 27.3°) maintaining summit depths less than 100 m. (**e**, **f**, **h**) are plots of the modelled probability of SICE with three different geophysical predictors: (**e**) summit depth, (**f**) average SST, and (**h**) degrees poleward are all significant predictors of SICE.

To explain which seamounts have chlorophyll enhancements while others do not, a binomial generalized linear model (GLM) was used to evaluate the probability of finding a significant enhancement (SICE) at a given seamount given its biogeophysical characteristics including summit depth and location (Table S1, see "Methods"). Seasonality and annual variability were also included in the model (see Table S2 for full models). This analysis revealed significant effects of summit depth and degrees poleward/long-term average SST (these predictors are highly correlated r = 0.92). Features with shallower summits depths and at lower latitudes (or higher average SSTs) were significantly more likely to induce significant chlorophyll enhancements (Fig. 2).

The fits from this binomial model are a bit more conservative than our summary statistics (Table 1), suggesting 33% of seamounts with summit depths around 100 m and 23% of seamounts with summit depths around 500 m will have SICE (see Fig. 2e, h).

### Seamount-induced chlorophyll enhancements can be persistent and high magnitude

The key argument against the seamount-induced chlorophyll enhancement hypothesis has been that they are ephemeral, rare, and low magnitude enhancements, which therefore cannot subsidize the food web^10^. However, our results indicate that SICE can increase decadal average chlorophyll values by up to 35% (average of 5%) relative to open ocean values in the validated Pacific subset (Fig. 2a) and 112% maximum (29% average) in the remote-sensing-based (unvalidated) global subset (Supplementary Information 1 Fig. 2). The signals are also evident at seasonal and monthly timescales, with the magnitudes of the enhancements and the variability in the magnitudes of the effect increasing with decreased temporal scales (Supplementary Movies S1 and S2). For example, the seamount with the largest magnitude effect in the validated subset, Robbie seamount (Fig. 2a), had a maximum monthly average enhancement of 56% relative to open ocean depths (February) and a sustained 4-month seasonal enhancement range of 40–56% between the months of December to March. Sustained enhancements of these magnitudes can subsidize the seamount ecosystem from the bottom-up because such timescales are sufficiently long (months to a decade) to allow for higher trophic level responses.

### Seamounts with chlorophyll enhancements have higher fisheries yields

Comparisons of historical fisheries catch data between seamounts with and without SICE revealed significantly higher yields at SICE seamounts (Fig. 1). All fisheries catch data are derived from the recently published global historical fisheries catch compilation gridded onto a 0.5° by 0.5° grid^20^ (see "Methods"). Total reported historical fisheries catch summed for each seamount area for the temporal extent of the fisheries dataset (1950–2015) was 2.2 times greater for SICE seamounts (mean catch 38,245 tonnes) than for non-SICE seamounts (mean catch 17,051 tonnes, *P* < 0.005, Supplementary Information 1 Tables 4 and 5). This included all fished marine taxa (N = 309) for the 177 seamounts in the Allain subset. Seamount fisheries are often short-lived, boom-bust fisheries, so a seamount may have a relatively low historical summed catch over the 64-year period but may have had a few years of intense, high yield fishing^21^. Therefore, we have also analyzed the maximum annual total catch for this seamount subset from all annual catches (see "Methods"). For a given seamount, this statistic represents the total catch for the seamount for its most productive fishing year. SICE seamounts also had a significantly higher mean maximum annual total catch (3,801 tonnes) compared to seamounts with no detectable chlorophyll enhancements (1842 tonnes; *P* < 0.005). Catch was also examined at the family level (Supplementary Information 1 Tables 4 and 5), which revealed that catch tends to be more diverse at SICE seamounts, and Scombridae (tunas, mackerels, and bonitos) is by far the number one caught family for both SICE and non-SICE seamounts.

## Discussion

Here we have presented the first evidence for long-term, persistent seamount-induced chlorophyll enhancements (SICE) of biologically significant magnitudes on basin and global scales. These results support the classic but disputed seamount paradigm of enhanced primary production and bottom-up food subsidies at seamounts^22^. Several papers have documented isolated in-situ observations of chlorophyll enhancements over specific seamounts^15,22,23^, and a recent study at Gorringe seamount (summit ~ 30 m) documents SICE both from simultaneous in-situ physical, chemical, and biological observations and satellite chlorophyll and SST data^24^. Nevertheless, a review of existing literature based primarily upon short-term oceanographic surveys concluded that enhanced phytoplankton over seamounts is a rare, short-lived phenomenon that can persist at most a few days at seamounts shallower than 300 m^10^. In contrast, our results suggest that SICE is evident in decadal mean surface chlorophyll, and strong seasonal enhancements can last for multiple months. Our results are also likely underestimating the true prevalence of SICE because our methodology is not capable of detecting sub-surface chlorophyll enhancements, which have been detected in oceanographic studies at specific seamounts such as Great Meteor Seamount^25^ or Minami-kasuga Seamount^22^ and linked to upwelling of deeper, colder waters^25^. Thus, if a large-scale time series of integrated water column chlorophyll-a were available for analysis, SICE would most likely be detected at a higher rate.

Though our analysis was primarily focused on the Pacific due to the spatial extent of the only published validated and cross-checked seamount database^16^, the Pacific contains nearly half of the world’s seamounts, and attempts at using a global unvalidated seamount database^2^ also found significant SICE in all oceans (Supplementary Information 1 Fig. 3). We suggest that previous ship-based studies did not have the spatial and temporal (greater than one month) sampling extent necessary to observe SICE, which led them to conclude that such effects are rare. Here we have shown clear seamount enhancements on timescales ranging from months to a decade, contradicting the idea that these phenomena are ephemeral and short lived. Finally, we have shown that chlorophyll enhancements can be up to 56% higher than proximate open ocean estimates. A 50–70% increase in primary production has been modeled to produce a four to ninefold increase in seamount fish biomass compared to a non-enhancement scenario^26^. Correspondingly, we have found a more than doubled fish catch at seamounts with chlorophyll enhancements (vs. seamounts without), strongly suggesting that SICE leads to food subsidies for commercially important species. Additionally, SICE seamounts account for a disproportionally high percent of the historical total summed fisheries catch of all analyzed seamounts: 23% of analyzed seamounts are responsible for 29% of the total catch. Our results also suggest a means to remotely evaluate and biologically classify seamounts to target ship-based research and inform resource managers. Now using remotely sensed chlorophyll data, or even just latitude and summit depth, we can identify those seamounts with a higher probability of SICE, and which likely have rich benthic and pelagic communities that could be high value targets for ecological research, conservation, and management.

Summit depth strongly influences the likelihood of detecting SICE at a given seamount. Though seamounts located near islands were removed from the models reported here, we did find that 80% of these seamounts were characterized by chlorophyll enhancements, connecting our findings on SICE to those of Gove et al. (2016) on the island mass effect, who reported that 91% of Pacific tropical/subtropical islands and atolls were ‘hotspots’ of phytoplankton biomass^19^. Here, we have extended this phenomenon of enhanced chlorophyll biomass to submerged features, whose summit depths are deeper than 30 m. The importance of summit depth was expected given the proposed physical mechanisms causing SICE^12,13^ (physical uplift of nutrient rich water), previously published satellite-based case studies^24,27^, and in-situ observations^28,29^. The shallower a given seamount extends, the more likely it becomes that the physical disturbances and mixing processes will bring additional deep, cool water rich in nutrients into the euphotic zone^13^. These physical processes are well documented in the physical oceanographic literature^12,13^. Cool, seamount-associated SST anomalies have been reported and linked to driving aggregations of pelagic fauna^24,30^. Our analysis of monthly average SSTs for the Allain seamounts subset yielded a significant negative correlation between SSTs and water depth, also supporting this idea that physical uplift of colder, nutrient rich waters over shallow topography may be the mechanism driving SICE. Additionally, our GAM analysis of SSTs found that the majority of seamounts had negative temperature-depth slopes. Though few of these relationships were statistically significant, this is likely due to the monthly timescale used in this analysis. Seamount-induced upwelling can be caused by internal wave pulses or tidal rectification^12^, which can occur as frequently as every several hours or daily. Such high frequency events would be difficult to detect with monthly averages and with 4 km spatial resolution. Moreover, while physical studies have shown upward doming of isopycnals and even the thermocline around seamounts^13,24,31,32^, few have shown that isopycnals to outcrop on the surface, so a temperature signal would not necessarily be detectable via satellite. An SST analysis across multiple time-scales in conjunction with in-situ water column measurements would be required to fully test the physical mechanism driving SICE.

The significance of summit location (specifically degrees poleward) to the probability of SICE detection is likely mechanistic. ‘Degrees poleward’ controls the inertial period through the Coriolis parameter resulting in longer inertial periods at low latitudes, meaning a larger range of oscillation frequencies will generate internal waves when interacting with a seamount^13^. The generation and reflection of internal waves at seamounts can increase turbulence, diapycnal diffusivity, and mixing at those seamounts^13^. These processes are able to influence waters well above the seamount summit and are therefore a possible mechanism increasing mixing and nutrient concentrations in the euphotic zone. For example, internal wave reflections have been documented at up to 750 m above bottom at Fieberling Guyot^33^. Therefore, the significance of seamount latitude could be due to changing internal wave dynamics.

Alternatively, temperature (highly correlated to degrees poleward) can influence stratification, and internal waves tend to be more energetic with increased stratification^33^. Low latitude seamounts with higher mean SSTs are generally in more intensely stratified waters, and in addition to more energetic internal wave dynamics, physical mixing processes in stratified areas may be more consequential in terms of adding nutrients to the euphotic zone at low latitudes versus at higher latitude seamounts in more well-mixed water. Mixed layer depth also tends to increase in depth with increasing degrees poleward^34^, and deep mixed layers can prevent phytoplankton blooms^35^. Unlike for euphotic depth, mixed layer depth was not explicitly included in our models, therefore perhaps some of the significant relationship between SICE occurrence and summit location may be explained by latitudinal trends in mixed layer depth. Finally, lower-latitude productivity tends to be nutrient limited whereas higher latitude productivity tends to be light limited, at least for part of the year^36^. Therefore, one would not expect to find a pronounced seamount effect on chlorophyll at high latitudes where phytoplankton are light limited. This may help to explain why, despite evidence of physical signatures (isopycnal doming and bottom current intensification) of seamount influence, only episodic chlorophyll enhancements have been observed at Cobb Seamount, a relatively high latitude seamount (46.7°N)^15^. Further work will be necessary to test these mechanisms, and all three of these processes may be at work simultaneously.

Our analysis of fisheries catch data around our Pacific seamounts has shown that SICE seamounts can provide double the fisheries catch. This analysis could be strengthened if detailed fisheries catch data became available that allowed for a comparison of catch from the same vessel on and away from a seamount. One could then calculate the increase in yield and directly compare the change in yield to the increase in chlorophyll at a seamount. Nevertheless, our analysis provides indirect evidence of a bottom-up food subsidy. Timescales required for bottom-up effects to reach top predators have been suggested at weeks to months^37^, and we have shown for the first time that SICE can persist on timescales up to a decade. Despite these results, a direct test of bottom-up seamount enhancements from chlorophyll to predator biomass is still missing, and a more detailed case study at a SICE seamount would be ideal for future work on this subject.

The results of this analysis have several further important limitations. While satellite chlorophyll data grants access to over a decade of monthly surface ocean color data, in-situ data for measuring chlorophyll concentrations to depth and net primary production are still missing. While we report surface chlorophyll-a concentrations, the data does not yet exist to allow for comment on seamount enhanced primary production, since increased chlorophyll is not exclusively attributable to increased production. However, the techniques presented here can be used to facilitate this next step by allowing researchers to efficiently target SICE-likely seamounts. Our analysis allows for the a-priori identification of seamounts which are most likely to have enriched benthic and pelagic communities and therefore be conservation and management targets.

## Methods

### Locating seamounts: seamount databases

Two seamount databases were used in this study: the validated Pacific database published by Allain et al. in 2008 (referred to in the text as the “Allain database”) and the most up-to-date global database published by Yesson et al. in 2011 (referred to in the text as the “Yesson database”). The primary analyses were conducted on a representative subsample of the Allain seamount database, the most spatially expansive (45°S–32°N and 130°E–120°W), validated and crosschecked published seamount database^16^. This area covers a large swath of the Pacific, which contains the vast majority of seamount features on our planet. Only “validated” seamounts, whose location and associated data were confirmed by at least one ship-based dataset rather than purely derived from satellite estimations, were used in the analyses. This subset was further reduced to include only features with validated summit depths deeper than 30 m (the optically shallow cutoff used after Gove et al. 2016) and elevations greater than 1,000 m (to follow the classic definition of a seamount as a feature rising more than 1,000 m above the seafloor). The resulting dataset was then subsampled to meet computational restrictions on database size. All seamounts with summit depths shallower than or equal to 300 m (48) were included, and the remaining features were subsampled such that 5 features were selected from each 100 m height bin and 1,500 m elevation bin for a total of 196 seamounts (of 485).

Second, to examine patterns globally, a subsample of the unvalidated Yesson database was analyzed with identical methodology. This database was based on the same global bathymetry used in this paper to derive underlying water depths for each chlorophyll pixel^1^. Only seamounts with estimated summit depths deeper than 30 m, elevations greater than 1,000 m, and estimated base areas greater than 500 km^2^ were selected from because the smallest features have the largest position and depth errors associated with them. Eight features were randomly sampled for each 150 m summit depth bin (ranging from − 30 to − 1,050 m) and from each 1,000 m elevation bin. These cutoffs were selected to create a subset of comparable size to the Allain subset and to maximize the chances of selecting from "real" features (those accurately detected via satellite and the Yesson seamount algorithm)^1, 2^. Because this database is unvalidated, these added precautions were taken in subset selection. The resulting subset (192 of 2,560) was then examined visually, summit depth estimates were corrected where needed, and features which were mistakenly identified as seamounts were removed from the subset. Despite our subsetting process, the manual revision still revealed problems with the published database, especially in estimated summit depth and location; therefore, approximately 19% of the initially selected seamounts had to be excluded from the final global analysis (final included number of seamounts = 166).

### Quantifying chlorophyll-a enhancements around seamounts

Chlorophyll-a (mg/m^3^) data were derived from the August 2015 version of the level 3 monthly composite, scientific quality, 0.0417° squared (~ 4 km) Moderate Resolution Imaging Spectroradiometer (MODIS) data (https://oceancolor.gsfc.nasa.gov/). Data were accessed through the NOAA ERDDAP, griddap site (https://coastwatch.pfeg.noaa.gov/erddap/griddap/erdMH1chlamday.html). A decade’s worth of chlorophyll data (Jan 2006-Jan 2016) were analyzed around each feature for a seamount-centered square with 100 km sides. Though seamounts whose validated summit depths were shallower than 30 m were excluded from the dataset entirely, an additional 30 m pixel depth (data source described below) cutoff was applied to all chlorophyll data to avoid potential bias from optically shallow waters anywhere in the sampling area, following the methods of Gove et al.^19^. Additionally, to avoid confusing the island mass effect (IME) with SICE, all seamounts whose sample area included one or more pixels with satellite estimated depths were emergent (≥ 0) were labeled “Emergent”. For all reported analyses these features flagged as ‘emergent’ (N = 19) were removed before statistical anlysis. All analyses included temporal predictors to account for seasonality (month predictor) and annual variability (year predictor) in chlorophyll patterns.

### Sea surface temperature

To test for the occurrence of seamount uplifted water, monthly daytime SSTs on the same ~ 4 km resolution from the Aqua MODIS platform were also downloaded for each 100 km sided seamount box (https://coastwatch.pfeg.noaa.gov/infog/MH1_sstMask_las.html). This data is science quality data from the August 2015 reprocessing of the global Level 3, 11 km SST data.

### Geophysical drivers

Seamount locations (summit latitude, summit degrees poleward or absolute latitude, summit longitude) and seamount specific information (elevation above the surrounding seafloor and summit depth below sea level) were derived from the published seamount databases described above^2,16^. Seasonality and annual variability were also included in the model through the incorporation of month and year terms. Each of the predictors was included for their theoretical influence on primary producers around seamounts. Summit location (i.e. latitude, longitude, and degrees poleward—defined as the absolute value of latitude) can influence internal wave dynamics^13^, mixed layer depth^34^, and global productivity dynamics including light versus nutrient limitation on production ^38^. Whether a seamount enhances production may well depend upon the background or long-term average productivity of the area, and this may co-vary with latitude and average SST (oligotrophic gyres are warm) at the summit. Average euphotic layer depth may influence the depth that physical seamount effects would need to reach in order to influence phytoplankton production. Finally, seamount summit depth greatly influences circulation patterns at the feature^13^ and thus possibly nutrient injection into the euphotic zone. However, seamounts often have complex geomorphologies, and therefore a variety of measures of summit depth were included: the shallowest depth at summit, proportion of pixels with depths shallower than the average euphotic layer depth, and proportion of pixels shallower than 800 m.

Depth data were derived from the Shuttle Radar Topography Mission (SRTM30 PLUS) 30 arc-second global bathymetry grid, which combines high resolution (~ 1 km) ship-based bathymetry data with ~ 9 km satellite-gravity data^39^ (https://topex.ucsd.edu/WWW_html/srtm30_plus.html). For each selected seamount, bathymetry and chlorophyll data were analyzed from a square region centered on the given summit location measuring 100 km^2^. Previous research suggested that the island mass effect (IME) extends approximately 30 km from the shore of islands^19^, and that seamount effects can extend up to 40 km from the summit location^18^, therefore, a box extending 50 km from the seamount summit was selected in order to ensure that the entire feature and both seamount-influenced waters and the surrounding unmodified open ocean waters were included in the analyses. Depth was extracted for each chlorophyll pixel using the extrapolation methods in the NOAA marmap package (getdepth function)^40^. In addition, because summit depth uses data from only the single shallowest point on a complex feature, two further depth-based predictors were derived: the proportion of chlorophyll pixels with depths shallower than 800 m (an estimate for the daytime maximum depth of vertical migration) and proportion of pixels shallower than the average euphotic depth at the seamount summit location.

Monthly composite 4 km resolution euphotic depth (in meters) calculated from the Lee algorithm was obtained from the NASA ocean color data product Zeu (e.g.: A200600A20060012006031.L3m_MO_ZLEE_Zeu_lee_4km.nc). The data were downloaded for the same period (2006–2016) as the chlorophyll data for each pixel around each selected seamount feature. The proportion of pixels in the sample region shallower than or equaling the overall average euphotic layer depth was calculated for each seamount.

Decadal average sea surface temperature (SST) at the summit locations were derived from available monthly mean ARGO SST data for each seamount (https://apdrc.soest.hawaii.edu/dods/public_data/Argo_Products/monthly_mean). These are therefore in-situ measured temperatures. Only data from the shallowest depth bin were used to derive these long-term average SSTs.

### Statistical models and model selection

All statistical analyses were conducted using the software package R. To identify seamounts characterized by SICE, defined as a statistically significant increase of chlorophyll with shallowing depths, we fit a Gaussian GAM for each seamount in each dataset analyzed. These models use the natural log of chlorophyll as the response and include a spatial predictor (two-dimensional relative latitude and longitude smoother), and a temporal predictor (month) to account for spatial and temporal autocorrelation respectively. Because phytoplankton are naturally patchy throughout the ocean, we included a two-dimensional spatial smoother to detect and account for this natural spatial structure. This approach made it possible to distinguish between depth related chlorophyll enhancements and random patchiness. An alternative approach might be to randomly select a control region away from the seamount for comparison. However, chl-a enhancements are likely to be asymmetrical and background levels are inherently patchy^19,41–43^. Our approach implicitly controls for such patchiness by testing for increases in chl-a with shallowing depth in a seamount-centered region that spreads well beyond the radius of any measured seamount effect, creating a control region that forms a ring around the region of interest instead of a single offset control region whose different position within the larger latitudinal and longitudinal spatial gradients in chlorophyll concentrations could skew the analysis^18,19^. Gove et al. (2016) took a very similar approach to their analysis of the island mass effect. These GAMs also fit a slope for each seamount between chlorophyll and depth using the decade of chlorophyll data for each corresponding sample area (see Supplementary Information 1 Table 1 for all full model formulas). The seamounts for which the resulting chlorophyll/depth estimate (seamount-specific slopes) were significantly positive (*P* < 0.05), were identified as SICE seamounts. Identical statistical techniques were applied to the SST analysis such that a GAM was used to describe seamount specific SST-depth slopes. In addition, correlations between SST and depth, SST and chlorophyll, and significantly negative SST slopes and SICE were estimated via Spearman correlations which assess the strength and direction of a monotonic relationship between two variables.

Finally, to test which predictors influence the likelihood of finding a significant seamount-induced chlorophyll enhancement as defined above, a binomial (or logit) GLM was fit. This model uses the presence/absence of SICE (determined by the results of the previously described GAM) as the binomial response variable and all available seamount-specific terms as predictors. These predictors are listed and fully described in Table S1: degrees poleward, summit latitude, summit longitude, summit depth, summit elevation, long-term average SST at the summit, decadal average chlorophyll, the standard deviation of the average chlorophyll, average euphotic depth, proportion of chlorophyll pixels with depths shallower than 800 m and shallower than the average euphotic depth, and a categorical variable identifying whether or not there is an emergent feature within the seamount sampling area (Table S1). Collinearity amongst the predictors was assessed via the variance inflation factor (VIF via the car package)^44^. Following the method by Zuur et al. 2010, collinearity was reduced using an iterative procedure whereby the predictor with the largest VIF was removed until all VIF estimates were below three^45^. Models of all possible combinations of the remaining predictors were then compared and ranked using the corrected Akaike’s information criterion (AICc in the MuMIn packag)^46^. The ‘best’, equivalent models, (those with AICc scores within 2 of the lowest AICc model), were evaluated and the frequency of significance of each predictor was tallied. Those predictors, which were significant across all ‘best’ models, were reported (Supplementary Information 1 Tables 1 and 2). Equivalent methods were also applied to the subset of seamounts from the global seamount database (N = 166).

The magnitudes of SICE were evaluated using similar techniques to those described previously for the logit model. However, the response was the magnitudes of the significant regression slopes. Models had low explanatory powers, non-uniform residuals for all applicable error distribution families, and no consistently superior model performance relative to null model. Therefore, instead of a statistical analysis of the magnitudes, those seamounts with the most positive slopes (the most extreme cases of SICE) were investigated in detail for the percent increase of chlorophyll over the seamount relative to surrounding surface waters over at least 4,000 m depth, over the entire 10-year period, as well as by year and by month. This gave a maximum enhancement magnitude and an evaluation of the seasonality and persistence of seamount-induced chlorophyll enhancements.

### Seamount fisheries catch analyses

To evaluate possible bottom up subsidies from SICE, historical seamount fisheries yields between the years of 1950 and 2015 were compared between those seamounts with significant chlorophyll enhancements and those without using data from Watson et al. 2018, a compilation of 15 different fisheries databases^20,47^. This database provides taxon-specific catches (ranging from class to species in taxonomic certainty) in tonnes mapped into half degree resolution cells by year, fishing country, and gear type broken into reported, illegal/unreported, and discards for both industrial and non-industrial fishing^20,47^. Only reported catch was analyzed here, but both industrial and non-industrial catch were included. Total reported catch was extracted for all fishing cells that overlapped partially or totally with any of the 177 seamount areas in the Allain database. Catch was prorated by the fraction of overlap between each fishing cell and the seamount area in question (e.g. if only 50% of the spatial area of a given cell overlapped with a given seamount area, catch from that fishing cell was multiplied by 0.5). Historical total reported fisheries catch (tonnes) was then calculated for each of the 177 seamounts (only those with no emergent pixels within their area) and compared between SICE and non-SICE seamounts using a generalized linear model with a Gamma distribution. In addition, maximum annual catch was calculated for each seamount because seamount fisheries are often characterized by boom-bust cycles, where fishing once commencing quickly ramps up, overexploits and decimates vulnerable stocks, and soon after returns to low levels as fishers move on to another fishing ground^7,9,48^. We define maximum annual catch as the total reported catch for a given seamount for its best fishing year on record. Again, maximum annual catch was compared between SICE and non-SICE seamounts using a generalized linear model with a Gamma distribution. All full model equations and model results are given in Supplementary Information 1 Table 3. Normality of residuals was evaluated visually using quantile–quantile plots, and residual variance was checked for similarity across groups. To further explore differences in catch between SICE and non-SICE seamounts, we also compared family-level catch between the two seamount categories by placing each taxa (309 unique taxa) in the database into its appropriate family and summing catch by family (see Supplementary Information 1 Tables 4 and 5 for a breakdown by SICE/non-SICE for families making up more than 1% of the catch).

## Supplementary information

Supplementary Information 1.

Supplementary Movie S1.

Supplementary Movie S2.

## Data Availability

All data used in this study are already publicly available from the repositories cited within this paper. However, the subsets of data specifically analyzed in this paper are also available by request from the lead author. All R code used to compile the datasets involved and run statistical analyses are available by request from the lead author as a .R file.
