# Supplementary material for: Evidence for long-term seamount-induced chlorophyll enhancements
Source: Sci Rep. 2020 Jul 29;10:12729. doi: 10.1038/s41598-020-69564-0 (PMC7391630; doi:10.1038/s41598-020-69564-0)
Supplement: Supplementary file 1 — Supplementary Information 1. [file 41598_2020_69564_MOESM1_ESM.pdf]

Evidence for Long-term Seamount-Induced Chlorophyll Enhancements

Authors: Astrid B. Leitner[1,2*], Anna B. Neuheimer[3], Jeffrey C. Drazen[2].

Affiliations:

[1] Monterey Bay Aquarium Research Institute, California

[2] Department of Biological Oceanography, School of Ocean Earth Science and Technology, University of Hawaii, Manoa.

[3] Aarhus Institute of Advanced Studies, Aarhus University, Denmark

*Correspondence to: aleitner@mbari.org

Supplementary Materials:

Supplementary Figures

Supplementary Tables

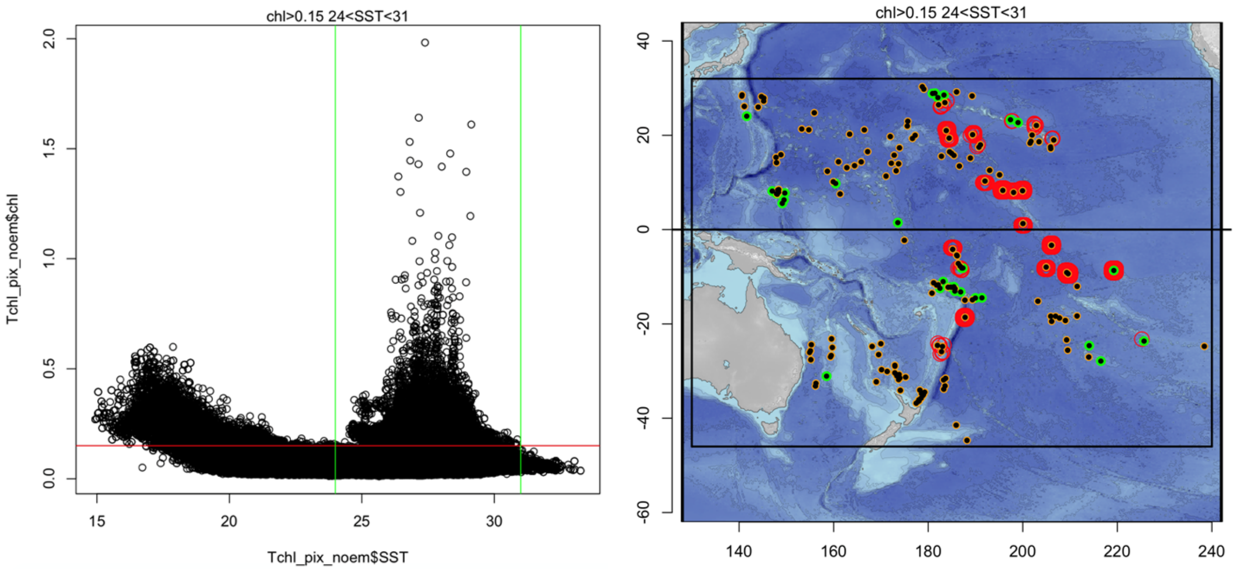

Fig. S1.

Relationship between chlorophyll and SSTs for those seamounts analyzed from the Allain dataset. Pixel by pixel SST versus pixel by pixel chlorophyll (left). Red line indicates chl=0.15 mg./m^3 which was taken as the lower limit for the unexpectedly high chlorophyll values between 24° and 31°C (marked by green lines). Red circles on the map of the Allain subset (Right) show the geographical location of these high chlorophyll, high temperature pixels. Figure and map generated using R[42] version 3.5.1 with package marmap[41].

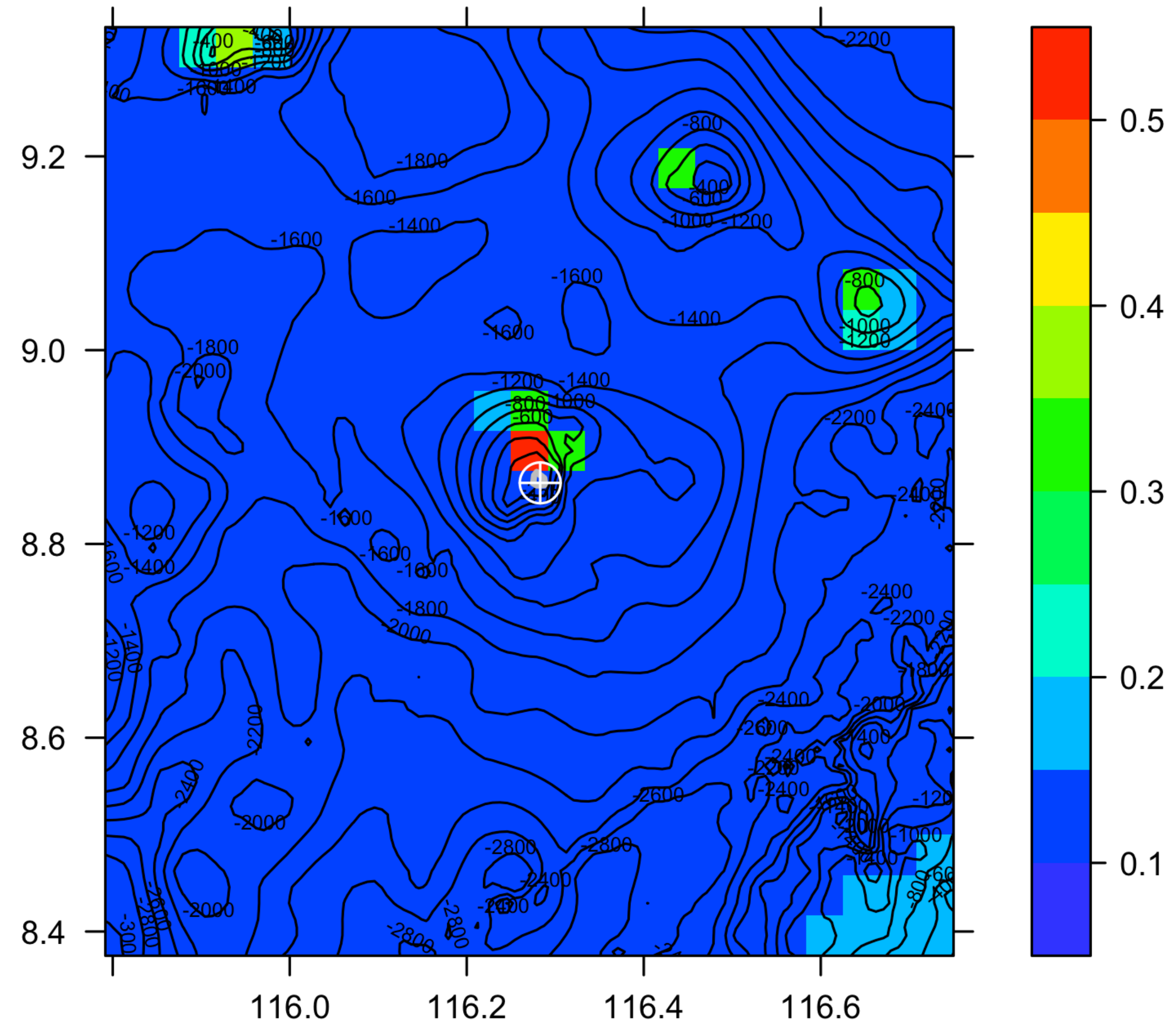

Fig. S2.

The 10-year average chlorophyll at Seamount Y3681803. This represents the maximum slope found in this dataset between depth and chlorophyll (112% increase from 4000 m to 30 m). Bathymetry is overlain by colors representing decadal per-pixel average chlorophyll-a concentrations in mg/m3. The white target symbol highlights the summit of the main feature of interest. The grey line represents the 30 m contour. Figure and map generated using R[42] version 3.5.1 with packages marmap[41] and lattice[51].

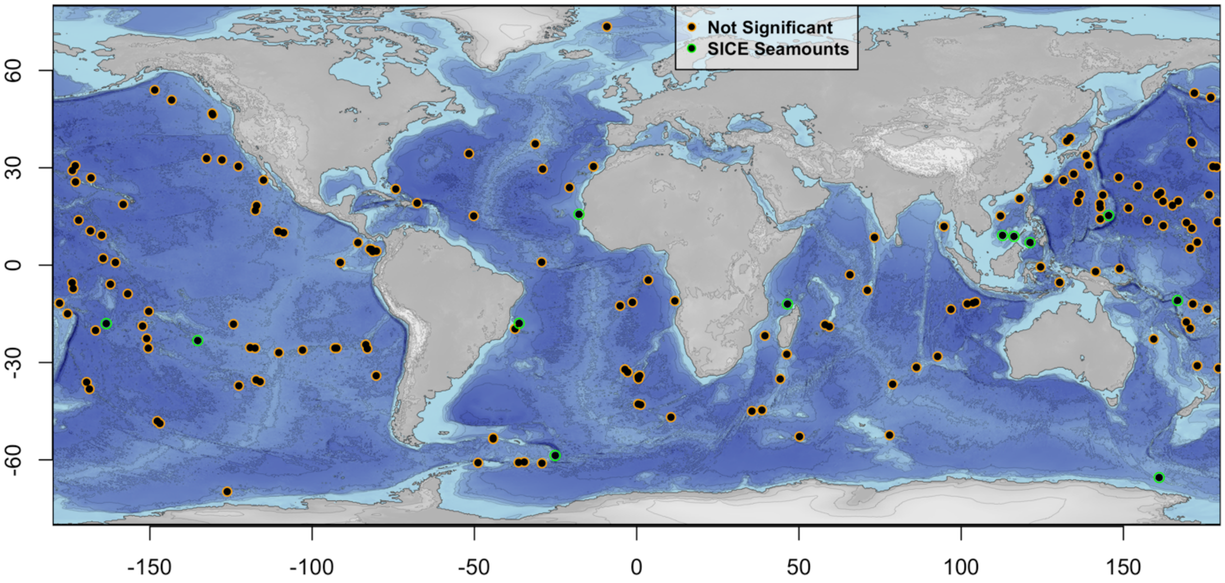

Fig. S3.

Map of the spatial extent of the global Yesson seamount database used in this study. Black dots represent the subsets of seamounts included in the analyses (166). Green outlines highlight those seamounts with significant seamount-induced chlorophyll enhancements (SICE), orange differentiates those without. Note this seamount is unvalidated since it is derived solely from remote-sensing techniques and sampled from the database published in Yesson et al. 2011. Figure and map generated using R[42] version 3.5.1 with package marmap[41].

Table S1.

Summary of geophysical predictors used in the statistical models.

Abbreviation	Full Predictor Name	Motivation for Inclusion
relLong, relLat	longitude and latitude relative to seamount summit position	To account for spatial autocorrelation via a 2-D spatial smoother
Month	month from which chlorophyll data stems	To account for temporal (monthly) autocorrelation
pixdepth	water depth underlying each chlorophyll pixel	To test if chlorophyll increases at shallower water depths (a main hypothesis)
Zeu	10-year average euphotic zone depth of seamount region	To account for global chlorophyll patterns
SD_chl	standard deviation of chlorophyll values of seamount region	To estimate the intensity of chlorophyll seasonality
DegFromEq	degrees poleward, absolute value of latitude; NOTE: highly correlated to SST	To estimate influences of internal wave dynamics, density stratification, light versus nutrient limitation for primary producers
Elevation	elevation of summit above surrounding seafloor	To link observed effects to physical mechanisms via a principle determining factors of the physics around seamounts (Lavelle and Mohn 2010)
SUMMIT	summit depth below sea level	To link observed effects to physical mechanisms via a principle determining factors of the physics around seamounts (Lavelle and Mohn 2010)
SummitLong360	summit longitude in 360 degrees east	To account for global chlorophyll patterns
SummitLat	summit latitude in +/- degrees	To account for global chlorophyll patterns
prop800	proportion of pixels of the seamount region with depths shallower than 800 m	To allow for complex seamount morphology. Seamount morphology may not be best characterized by summit depth, a single point; 800 m is an approximate daytime depth for the deep scattering layer
LT_avgchl	long term (10 year) average chlorophyll for the seamount region	To allow for differences in background (open-ocean) chlorophyll concentration. SICE may not be noticeable in highly productive regions since the production is already high
propZeu	proportion of pixels of the seamount region with depths shallower than the average euphotic zone depth of the seamount region for the sample period (one decade)	To allow for complex seamount morphology. Seamount morphology may not be best characterized by summit depth, a single point; primary production occurs only in the euphotic zone; if the seamount itself reaches into the euphotic zone physical effects will influence primary producers
SST_summit	A decadal average sea surface temperature at summit position	To link observed effects to physical mechanisms. Temperature influences stratification (determined by temperature, salinity, and pressure), and local density

Table S2.

Final Statistical Models Used. Full model formulas are given for each model fit in this analysis along with a general description of model type and each model's purpose.

Formula	Description	Purpose
bam(log(chl) ~ pixdepth + Seamount + pixdepth : Seamount + te(relLong, relLat, by=Seamount) + s(Month, by=Seamount, bs="cr", k=10), family= gaussian, data= SMdb, cluster=cl, gc.level=2)	large Gaussian GAM	fit a chlorophyll-depth relationship for each seamount (Allain subset)
glm(signif ~ DegFromEq+Emergent+SUMMIT, data=slopesdata, family="binomial", na.action="na.fail")	logit GLM, chosen model after model selection	examine which seamount predictors effect presence or absence of SICE for Allain Seamounts
bam(log(chl) ~ pixdepth + Seamount + pixdepth : Seamount + te(relLong, relLat, by=Seamount) + s(Month, by=Seamount, bs='cr', k=10), family= gaussian, data= SMdb, cluster=cl, gc.level=2)	large Gaussian GAM	fit a chlorophyll-depth slope for each seamount global subset)
glm(signif ~ Elevation+Emergent+SD_chl+propZeu+SUMMIT, data=slopesdata, family="binomial", na.action="na.fail")	logit GLM, chosen model after model selection	examine which seamount predictors effect presence or absence of SICE for global subset

Table S3.

Model selection criteria and explanations for the logit model for presence or absence of SICE for Allain subset. This table details formulas for each of the "best" AICc equivalent models. Frequency of significance is tabulated for each predictor that was significant in at least one best model (given in number significant/total best models). Finally, the model chosen for drawing plots in Figure 2 is presented.

Best Models:

**AICc**	**Model Formula**	**delta AICc**
143.5299	signif ~ DegFromEq+SummitDepth	0
144.124	signif ~ DegFromEq+LT_avgchl+SummitDepth	-0.594
144.1545	signif ~ DegFromEq+SummitDepth+Zeu	-0.625
144.4752	signif ~ DegFromEq+Elevation+SummitDepth+SummitLat+SummitLong360	-0.945
144.6294	signif ~ DegFromEq+SummitDepth+SummitLong360	-1.099
144.7633	signif ~ DegFromEq+SummitDepth+SummitLat	-1.233
144.9207	signif ~ DegFromEq+SummitDepth+SummitLat+SummitLong360	-1.391
145.2174	signif ~ DegFromEq+Elevation+SummitDepth	-1.687
145.4051	signif ~ DegFromEq+SummitDepth+SummitLong360+Zeu	-1.875
145.4383	signif ~ DegFromEq+LT_avgchl+SummitDepth+SummitLong360	-1.908
145.4433	signif ~ DegFromEq+Elevation+LT_avgchl+SummitDepth	-1.913
145.5033	signif ~ DegFromEq+Elevation+SummitDepth+Zeu	-1.973

**Predictor**	**Frequency of Significance in Best Models**
DegFromEq	12/12
SummitDepth	12/12

Chosen Model:

```
Call: glm(formula = signif ~ DegFromEq + Elevation + SummitDepth +
    SummitLat + SummitLong360, family = ModelFamily, data = myData,
    na.action = "na.fail")

Deviance Residuals:
    Min       1Q   Median       3Q      Max
-1.4262  -0.5893  -0.3713  -0.1875   3.0031

Coefficients:
            Estimate Std. Error z value Pr(>|z|)
```

```
(Intercept)   -2.6353543  2.1201853  -1.243 0.213874
DegFromEq     -0.0700937  0.0262423  -2.671 0.007562 **
Elevation     -0.0003412  0.0002250  -1.517 0.129368
SummitDepth   -0.0011727  0.0003114  -3.765 0.000166 ***
SummitLat      0.0257971  0.0140627   1.834 0.066590 .
SummitLong360  0.0251057  0.0131705   1.906 0.056625 .
---
Signif. codes:  0 '***' 0.001 '**' 0.01 '*' 0.05 '.' 0.1 ' ' 1

(Dispersion parameter for binomial family taken to be 1)

    Null deviance: 161.10  on 176  degrees of freedom
Residual deviance: 131.98  on 171  degrees of freedom
AIC: 143.98

Number of Fisher Scoring iterations: 6
```

Table S4.

Models and model results from the fisheries analyses. Data is from the Watson et al. 2018 fisheries database. Models only use the subset of the entire seamount dataset which have no emergent pixels in their sample areas (N=177). The 'Mean' column shows the mean of the response variable (in tonnes) for SICE seamounts (above in bold) and for non-SICE seamounts (below).

Response	Equation	Mean	P-value
**Historical Total Catch (1950-2015)**	glm(formula = TotalRepCatch_Tonnes_UofT ~ signif, family = Gamma, data = sminfo_noIME	**38245.38**<br>17050.61	**0.000903 *****
**Maximum Total Annual Catch (single yr)**	glm(formula = MaxAnRepCatch_Tonnes_UofT ~ signif, family = Gamma, data = sminfo_noIME)	**45288.24**<br>24858.03	**0.0033 ****

Table S5.

Historical total family catch from the Watson et al. 2018 database grouped by SICE and Non-SICE seamounts from the Allain seamount subset excluding seamounts with one or more emergent cells in the seamount area (N=177). Families making up more than 1% of the total historical catch at each SICE and Non-SICE seamounts. Total area included in the SICE and Non-SICE categories are given in square kilometers next to the section heading. Families are ordered by total family catch (given in tonnes). Family catch standardized by square kilometer is also given for comparison. Finally percent of total historical catch is presented for each family for both the SICE and non-SICE seamount groups.

	Family	Total Family Catch	Family Catch per km2	% Catch
**SICE: 300416.4 km sq**				
	Scombridae	486098.47	1.618	42.37
	Marine fishes	191218.72	0.637	16.67
	Trichiuridae	47671.35	0.159	4.15
	Teuthida	32226.88	0.107	2.81
	Carangidae	25961.45	0.086	2.26
	Scorpaenidae	25466.39	0.085	2.22
	Miscellaneous marine crustaceans	24463.36	0.081	2.13
	Salmonidae	19435.93	0.065	1.69
	Clupeidae	18998.85	0.063	1.66
	Octopoda	18771.72	0.062	1.64
	Lethrinidae	17620.53	0.059	1.54
	Mugilidae	16182.66	0.054	1.41
	Berycidae	13978.85	0.047	1.22
	Istiophoridae	13307.59	0.044	1.16
	Pleuronectiformes	12957.73	0.043	1.13
	Hexagrammidae	12356.16	0.041	1.08
	Miscellaneous marine molluscs	12304.71	0.041	1.07
	Sepiida	12142.09	0.04	1.06
	Sharks, rays, chimaeras	11722.97	0.039	1.02
**NON-SICE: 1473458 km sq**				
	Scombridae	1749862.54	1.188	69.81
	Carangidae	205875.95	0.14	8.21
	Marine fishes	155062.53	0.105	6.19
	Trichiuridae	58135.36	0.039	2.32
	Clupeidae	40946.34	0.028	1.63

Table S6.

Maximum annual family catch from the Watson et al. 2018 database grouped by SICE and Non-SICE seamounts from the Allain seamount subset excluding seamounts with one or more emergent cells in the seamount area (N=177). Families making up more than 1% of maximum annual catch (the catch at a given seamount for it's most productive fishing year) at each SICE and Non-SICE seamounts. Total area included in the SICE and Non-SICE categories are given in square kilometers next to the section heading. Families are ordered by total family catch (given in tonnes). Family catch standardized by square kilometer is also given for comparison. Finally, percent of total historical catch is presented for each family for both the SICE and non-SICE seamount groups.

	Family	Max Annual Family Catch	Family Catch per sq km	% CATCH
SICE: 300416.4 sq km				
	Scombridae	51018.95	0.17	44.74
	Marine fishes	34850.88	0.116	30.56
	Clupeidae	10485.68	0.035	9.19
	Haemulidae	4284.55	0.014	3.76
	Miscellaneous marine crustaceans	1663.38	0.006	1.46
	Hexagrammidae	1560.96	0.005	1.37
NON-SICE: 1473458 sq km				
	Scombridae	193778	0.132	71.55
	Marine fishes	26369.06	0.018	9.74
	Carangidae	17267.79	0.012	6.38
	Trichiuridae	13574.59	0.009	5.01
	Marine animals	3518.35	0.002	1.3
	Clupeidae	2829.35	0.002	1.04

Movie S1. Monthly Average Chl

Monthly average (over 10 year study period) chlorophyll concentrations around Hotspur Seamount (summit depth 30m, latitude 18°S). White pixels contained depths ≤ 30m and were removed to prevent incorporation of optically shallow waters into analysis.

Movie S2. Yearly Average Chl

Yearly average (over 10 year study period) chlorophyll concentrations around Hotspur Seamount (summit depth 30m, latitude 18°S). White pixels contained depths ≤ 30m and were removed to prevent incorporation of optically shallow waters into analysis.
